# Kinetic modeling and optimization of ethanol fermentation by the marine yeast *Wickerhamomyces subpelliculosus* ZE75

**DOI:** 10.1007/s11274-024-03942-y

**Published:** 2024-04-06

**Authors:** Heba Hawary, Abdel-Kareem M. Marwa, Abdel-Hamied M. Rasmey

**Affiliations:** 1 Botany and Microbiology Department, Faculty of Science, Suez University, Suez, 43221 Egypt; 2 Botany and Microbiology Department, Faculty of Science, Sohag University, Sohag, 82524 Egypt

**Keywords:** Biofuel, Ethanol fermentation, Marine yeast, Optimization, Kinetic study, *Wickerhamomyces subpelliculosus*

## Abstract

The study aims to enhance ethanol production by *Wickerhamomyces subpelliculosus* ZE75 isolated from marine sediment. In addition, analyzing the kinetic parameters of ethanol production and optimization of the fermentation conditions was performed. The marine yeast isolate ZE75 was selected as the front runner ethanol-producer, with an ethanol yield of 89.77 gL^−1^. ZE75 was identified relying on the phenotypic and genotypic characteristics of *W. subpelliculosus*. The genotypic characterization based on the Internal Transcribed Spacer (ITS) sequence was deposited in the GenBank database with the accession number OP715873. The maximum specific ethanol production rate (*v*_*max*_) was 0.482 gg^−1^ h^−1^ at 175 gL^−1^ glucose concentration, with a high accuracy of *R*^2^ 0.95. The maximum growth specific rates (*μ*_max_) were 0.141 h^−1^ obtained at 150 gL^−1^ glucose concentration with *R*^2^ 0.91. Optimization of the fermentation parameters such as pH and salinity has been achieved. The highest ethanol yield 0.5637 gg^−1^ was achieved in a 100% natural seawater-based medium. The maximum ethanol production of 104.04 gL^−1^ was achieved at pH 4.5 with a specific ethanol rate of 0.1669 gg^−1^ h^−1^. The findings of the present study recommend the possibility of ethanol production from a seawater-based medium on a large scale using *W. subpelliculosus* ZE75.

**Graphical abstract:**

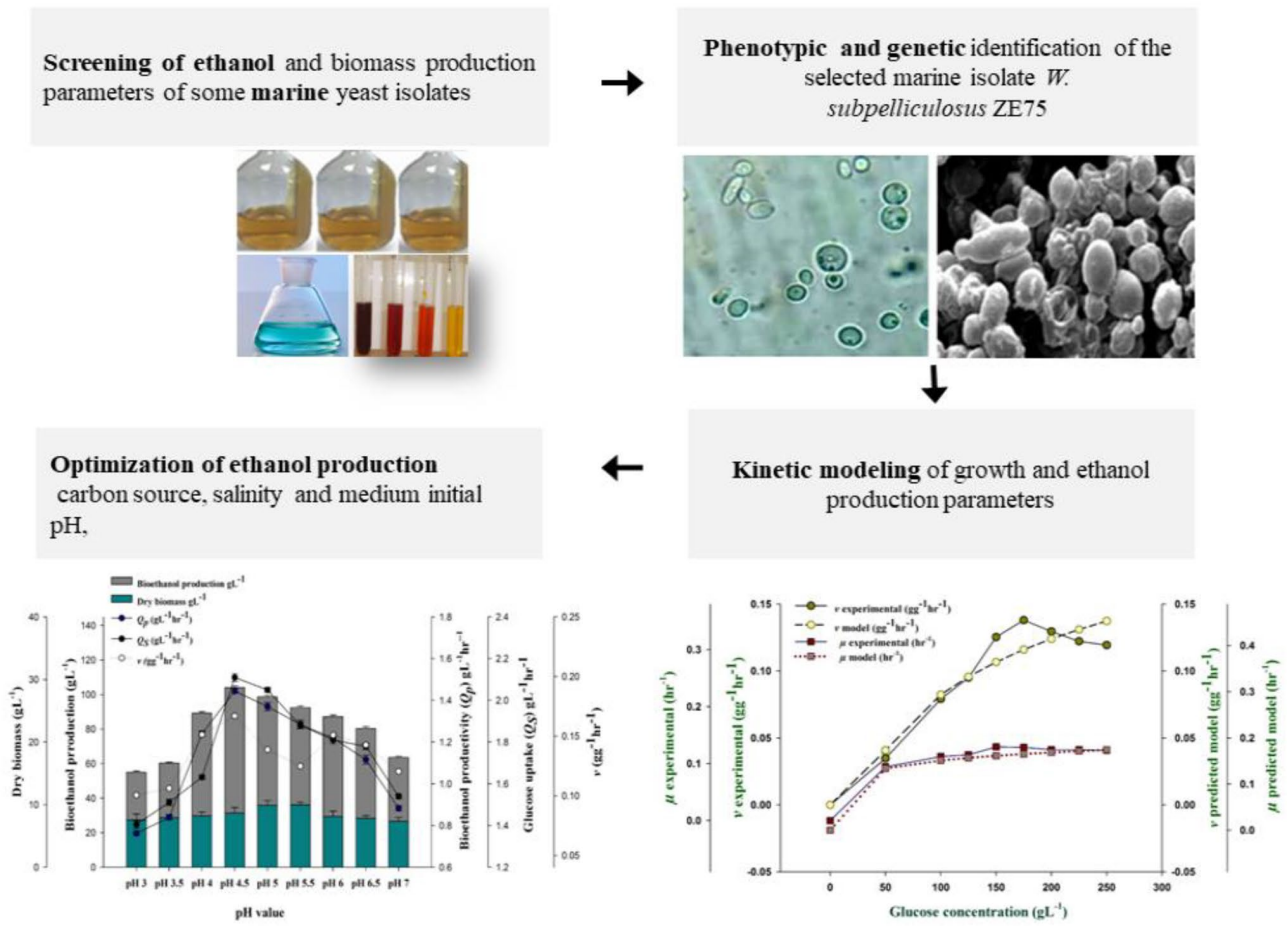

**Supplementary Information:**

The online version contains supplementary material available at 10.1007/s11274-024-03942-y.

## Introduction

Global soaring oil prices, climate change and ultimately the global energy crisis resulted from the Russian-Ukrainian war has alerted the world that it is necessary to hasten the transition to more sustainable and ecofriendly energy resources as alternatives to the currently used fossil energy fuel. Therefore, strong policies are required in the different countries of the world to encourage investment in renewable energy resources to reduce the risks of high energy prices and global warming. Biofuels have played a significant role as an eco-friendly option to meet the ever-increasing energy demand (Oves et al. 2022). In recent years, there has been a focus on the production and usage of liquid biofuels as promising substitutes for fossil fuels.

Bioethanol is the most applicable and promising of liquid biofuels that is characterized by sustainability, cleaning, and lower greenhouse gas emissions (Bušić et al. 2018). Furthermore, ethanol has become a crucial demand for various industrial purposes. It is an unusual organic oxygen-containing chemical with a unique set of properties as a solvent, drinkable, bactericide, antifreeze, fuel, inhibitor disinfecting agent, preservative, and intermediate for organic compounds (Brooks 2008).

Ethanol can be synthesized either by fermentation of sugars or by reacting ethene with steam. Synthetic production of ethanol has several disadvantages as it releases poisonous gases into the atmosphere, is made from nonrenewable sources, and has high energy consumption (Bhatia et al. 2012). Ethanol production through fermentation offers enhanced energy security and a more favorable trade balance (Bhatia et al. 2014).

There is still much research required to be done in terms of improving bioethanol technology pathways and obtaining new strains that are highly efficient in bioethanol production and can tolerate the inhibitors result during the fermentation process (Rasmey et al. 2017; Greetham et al. 2019a, b). Newly characterized strains with high fermentation rates can be achieved by altering significant genes in the bioethanol production pathway by exploring extreme habitats like marine water (Hawary et al. 2019).

Yeasts, especially marine yeasts, are extensively used for wine and fuel ethanol fermentations due to their various special features like high growth rates (anaerobically or aerobically), proficient ethanol fermentation, and capability to tolerate various stresses (Dashko et al. 2014). Marine yeasts are distinctive in fermenting a wide variety of substrates under high salt concentrations. Besides *Saccharomyces cerevisiae*, marine-derived yeasts like *Pichia*, *Yarrowia*, *Debaromyces*, *Wickerhamomyces,* and *Candida* species have also been investigated for their potential of bioethanol production (Zaky et al. 2014).

The unique characteristics of marine yeasts, particularly high osmotolerance and halotolerance, make them an ideal candidate for bioethanol production especially when using seawater instead of freshwater in the fermentation medium (Zaky et al. 2018). Furthermore, the use of seawater-based medium for bioethanol production could be a hopeful strategy for saving fresh water (Greetham et al. 2019a, b). The aim of the current study is to evaluate the potentiality of isolated marine yeasts for bioethanol production using a seawater-based fermentation medium. In addition, to investigate the impact of carbon, salinity, and pH of the fermentation medium on bioethanol productivity.

## Materials and methods

### Microorganisms source

The studied yeasts were isolated on yeast malt agar (YMA) from marine sediment samples collected from Suez Canal, Egypt. YMA is composed of (g/L seawater): yeast extract, 3.0; malt extract, 3.0; peptone, 5.0; dextrose, 10.0 and supplemented with 20.0 g agar–agar. The medium pH was adjusted to 5.5 ± 0.2 using 0.1 N HCl and 0.1 N NaOH.

### Inocula preparation

Yeast inoculum (1 × 10^6^ cells/mL) was prepared by inoculating a loopful of 48 h old culture to 50 ml YM broth and incubated on a rotary shaker (150 rpm) at 30 °C for 48 h.

### Fermentation process

The fermentation process was conducted on YM broth supplemented with 150 gL^−1^ (15%) glucose in 100 mL glass bottles containing 50 ml medium inoculated with 20% inocula. The cultures were incubated on a rotary shaker (150 rpm) at 30 °C for 5 h at aerobic conditions and continued to 48 h under anaerobic conditions.

### Ethanol determination

Ethanol (v/v) was assayed by potassium dichromate oxidation method according to Michałowska-Kaczmarczyk and Michałowski (2019).

### Total residual sugars (TRS) determination

The residual sugars in the spent fermentation medium were determined by dinitrosalicylic acid (DNS) method as described by Miller (1959).

### Biomass determination

The biomass was determined as dry weight (gL^−1^) by drying the collected cells at 85 °C for 24 h (Zhang et al. 2018).

### Kinetic analysis

Ethanol concentrations (*P*_*E*_), biomass (*X*_*E*_), ethanol coefficient yield (*Y*_*P/S*_), biomass coefficient yield (*Y*_*X/S*_), volumetric ethanol productivity (*Q*_*p*_) and average substrate uptake (*Q*_*s*_) were calculated based on the following equations. Ethanol and biomass coefficient yields were based on initial sugar concentration (S_Go_) and expressed in gg^−1^ [Eqs. 1, 2], volumetric ethanol productivity was calculated based on the actual ethanol concentration produced EP (g L^−1^) in the fermentation time t (hr) giving the highest ethanol concentration [Eq. 3] while the average substrate uptake was calculated as grams of substrate consumed per liter per hour (gL^−1^ h^−1^).

To determine the microbial kinetic growth, the specific ethanol production rates (*v)* and the specific growth rate (*µ*_*x*_) values were calculated. Specific ethanol production rates (*v*) were expressed as gg^−1^ h^−1^ and calculated from the following relationship by using the changes in ethanol and dry biomass concentrations with time. The specific growth rate values were calculated from the logarithmic plots of the dry weight data with the fermentation time. The maximum values of specific growth rates (*µ*_max_), maximum specific ethanol production rates (*v*_*max*_), maximum dry weight (*X*_*max*_) and the maximum ethanol concentrations (*p*_*max*_) were also calculated.

1 $$Y_{p/s} \left( {{\text{g g}}^{ - 1} } \right) = P_{E} /S_{G0}$$

2 $$Y_{x/s} \left( {{\text{g g}}^{ - 1} } \right) = X_{E} /S_{G0}$$

3 $$Q_{p} \left( {{\text{gL}}^{ - 1} {\text{hr}}^{ - 1} } \right) = dEP/dt$$

4 $$Q_{s} \left( {{\text{gL}}^{ - 1} {\text{hr}}^{ - 1} } \right) = - dS/dt$$

5 $$\it v \, ({\text{gg}}^{{ - {1}}} {\text{hr}}^{{ - {1}}} ) = dP/{\text{X}}dt$$

6 $$\mu_{x} = dX/dt$$

### Phenotypic characterization

#### Macro and micro-morphological characteristics

Colony characteristics (color, texture, appearance, elevation, and margin) were examined in cultures streaked on YMA. The cell morphology (budding, ascospores, and pellicle) was examined in 5% malt broth medium (Vaughan-Martini and Martini 1993) and sodium acetate agar medium (Sulieman, et al. 2015). Pseudohyphae were visualized on cornmeal agar using the coverslip method (Kurtzman et al. 2011).

#### Biochemical characteristics

Diazonium blue B (DBB) test, amylase production, cellulase production, urease production, citrate test, indole production, methyl red (MR), Voges-Proskauer (VP), growth in vitamin-free and osmotic medium, assimilation and fermentation of various carbon compounds were studied according to Kurtzman et al. 2011). Also, the temperature and halo-tolerance profiles of the growth were conducted.

#### Genotypic identification

Genomic DNA (gDNA) was extracted using chloroform-extraction and ethanol-precipitation method (Kumar et al. 2010). The primers ITS1:5′-TCCGTAGGTGAACCTGCGG-3′and ITS4 5′ TCCTCCGCTTATTGATATGC-3′ were used to amplify ~ 750 bp from the Internal Transcribed Spacer (ITS) Region. PCR reactions were elevated in a final volume of 100 µL with the following reagent concentrations: Taq buffer (1x); dNTP mixture (200 µM each); Forward and reverse primers (0.2 µM each); Taq DNA polymerase (2.5 U/100 µL); ~ 50:100 ng of gDNA template and the final volume of the PCR reaction adjusted to 100 µL with nuclease-free H_2_O. PCR amplification had an initial denaturation step at 95 °C for 3 min, followed by 35 cycles: 95 °C for 30 s, annealing at 55 °C for 30 s, extension 72 °C for 60 s, and a final extension at 72 °C for 5 min. The amplified PCR product was sent to Solgent Co Ltd (South Korea) for sequencing. The resulting sequences were trimmed and assembled in Geneious software (Biomatters). Consequently, the trimmed sequences were identified by search in the basic local alignment tool (BLAST) in GenBank. The full-length sequences obtained were matched with previously published sequences available in NCBI using BLAST at NCBI website: http://www.ncbi.nlm.nih.gov/BLAST/ to assess the degree of DNA similarity.

#### Optimization of ethanol production

Effects of carbon source, salinity, and medium initial pH were studied to obtain the maximum yield of ethanol. The fermentation medium was supplemented individually with organic different carbon sources (glucose, fructose, sucrose, maltose, and lactose) at a concentration of 15% of each carbon source individually. The effect of different salinity concentrations (0.5M, 1M, 2M, 3M, 4M, seawater, 50% seawater and distilled water) on ethanol production was studied. The effect of initial pH (3–7 with 0.5 interval) was tested.

### Statistical analysis

Ethanol production and biomass mean values were compared at 5% significance level using Tukey’s test. Non-linear regression analysis was evaluated using SPSS 10 statistical package program.

## Results

### Screening of bioethanol and biomass production by the marine yeast isolates

Results in Table 1 indicated that all the tested yeast isolates (ZE2, ZE15, ZE68, ZE5, and ZE102) could produce ethanol at variable concentrations using a seawater-based medium. The highest ethanol production (89.77 gL^−1^) was produced by the isolate ZE75 with volumetric ethanol productivity of 1.247 gL^−1^ h^−1^. On the other hand, the isolate ZE produced the lowest ethanol production (59.019 gL^−1^), with volumetric ethanol productivity of 0.819 gL^−1^ h^−1^.

**Table 1:** Ethanol and biomass yield by five yeast isolates

Parameters	ZE2	ZE15	ZE68	ZE75	ZE102
Ethanol, *E*_*p*_ (gL^−1^)	69.36^a^ ± 0.2	84.48^b^ ± 0.1	60. 69^c^ ± 0.09	89.77^d^ ± 0.17	59.019^C^ ± 0.1
Biomass, *X*_*m*_ (gL^−1^)	7.49^a^ ± 0.22	6.944^b^ ± 0.14	7.099^b^ ± 0.21	9.044^c^ ± 0.31	5.554^d^ ± 0.25
Volumetric ethanol productivity, *Q*_*p*_ (gL^−1^h^−1^)	0.9633	1.1733	0.843	1.247	0.819
Specific ethanol production rates, *v*_*g*_ (gg^−1^h^−1^)	0.129	0.169	0.119	0.138	0.147
Ethanol coefficient yield, *Y*_*P/S*_ (gg^−1^)	0.4624	0.563	0.4046	0.598	0.393
Biomass coefficient yield, *Y*_*X/S*_ (gg^−1^)	0.0499	0.0463	0.0473	0.066	0.037
Average glucose uptake, *Q*_*s*_ (gL^−1^h^−1^)	2.00	2.30	2.31	2.06	2.18

Values are means of three replicates; values followed by same letters on the same row are not significantly different (P < 0.005) in Tukey’s test

### Phenotypic characterization and genotypic identification of the isolate ZE75

**Fig. 1:**
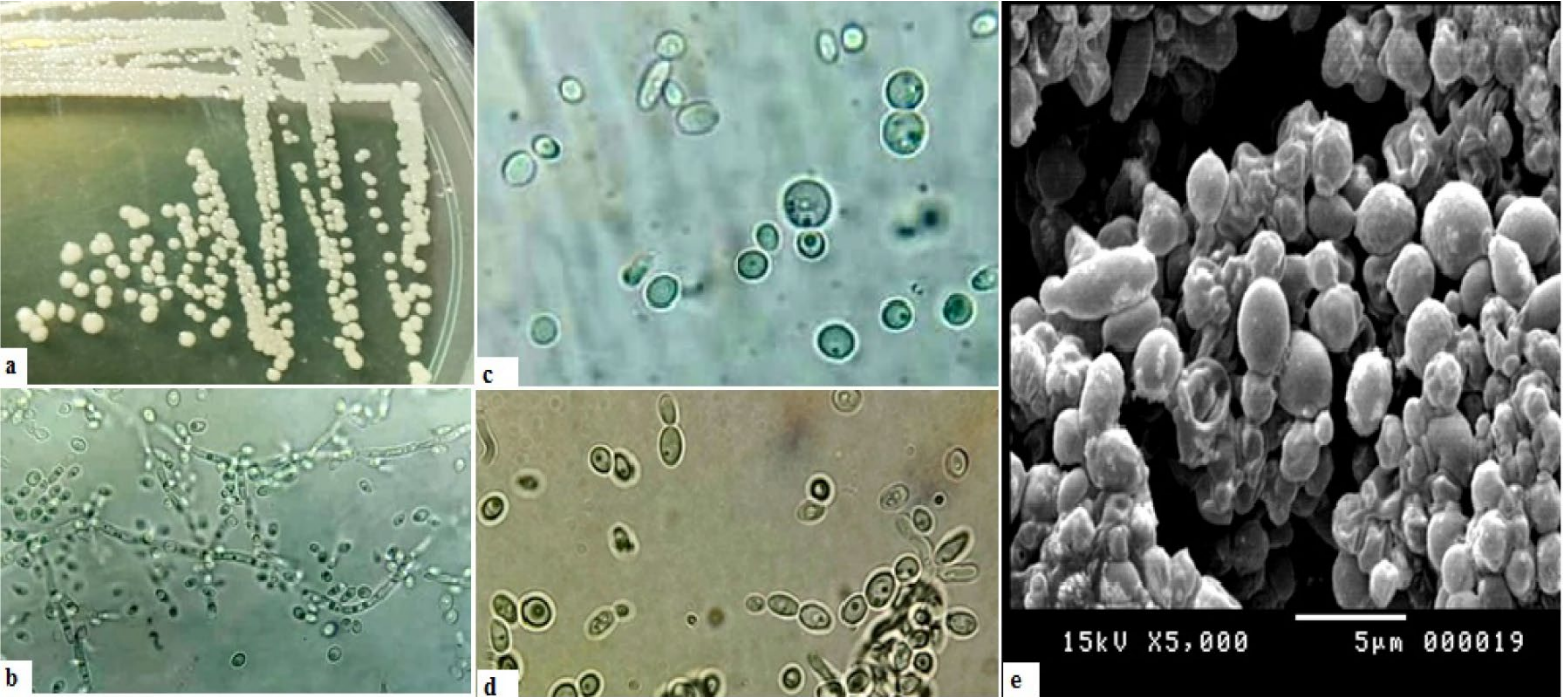
Morphological characteristics of the yeast isolate ZE75: **a** The yeast colonies on YM agar were tannish- white colored, butryous, smooth, glistening and convex, **b** Pseudohyphae formation on cornmeal agar (×40), **c** Cells are subglobose, asci containing two to eight hat shaped ascospores on on sodium acetate agar (×100), **d** Chains of subglobose to ovoidal cells have monobolar budding on 5% malt broth (×100), **e** Scanning electron microscope (SEM) of pseudohyphae formation on cornmeal agar (×3.500)

The colony characteristics of the isolate ZE75 are shown in Table 2. The colony on YM agar was tannish-white colored smooth, butryous, glistening, and convex. Cells’ morphological characters were measured on 5% Malt extract broth and sodium acetate agar media; it was revealed that the cell shape was sub-globose to ovoidal with monopolar budding. Diploid budding cells are transformed into asci containing two to eight hat-shaped ascospores. Pseudohyphae were formed on cornmeal agar. Pseudohyphae were branching out that have ramified chains of cells bearing dense clusters of ovoidal ballistoconidia in verticils, the cells are transformed into asci with ascospores (Fig. 1). The physiological and biochemical characteristics (S1, supplementary file) revealed that the isolate ZE75 was ascomycetous yeast and able to hydrolyze cellulose and utilize glucose, sucrose, maltose, lactose, erythritol, L-arabinose, cellobiose, nitrate, and glycerol, but not D- xylose and nitrate. It couldn’t assimilate or ferment galactose, negative to Voges-Proskauer (VP), indole tests and methyl red (MR). Furthermore, results also indicated that these nonconventional ascomycetes yeast was able to grow at 50% glucose and vitamin-free medium. It could tolerate high sodium chloride concentrations up to 4.0 M and grow well at a temperature range 8–42 °C.

**Table 2:** Phenotypic characteristics of *W. subpelliculosus* ZE75

Medium	Character	Observation
Yeast malt agar	Colony color	Tannish- white
	Nature	Butryous
	Appearance	Smooth, glistening
	Elevation	convex
	Margin	Entire
5% Malt broth	Cell shape	Subglobose to ovoidal
	Budding	Monobloar
	Sporulation	Diploid budding cells are transformed into asci containing two to eight hat shaped ascospores
	Pellicle formation	Formed and thick folded
Sodium acetate agar	Cell shape	Subglobose to ovoidal
	Budding	Monobloar
	Sporulation	Asci containing two to eight hat shaped ascospores
	Conjugation	–
Cornmeal agar	Pseudohyphae formation	Pseudohyphae are branching out that have ramified chains of cells bearing dense clusters of ovoidal ballistoconidia in verticils, the cells are transformed into asci with ascospores

The genotypic identification of the isolate ZE75 was relying on phylogenetic analysis. By the comparison of ITS-rDNA gene sequence (583 bp) of the isolate and ITS-rDNA gene sequences of the GenBank database, the isolate was genetically identified as *Wickerhamomyces subpelliculosus* with a similarity of 99.48% with *Wickerhamomyces subpelliculosus* MK156309. The novel isolate was deposited under the accession number OP715873 in the GeneBank. The isolate taxonomic position was family Phaffomycetaceae, order Saccharomycetales, subphylum Saccharomycotina in the phylum Ascomycota. The phylogenetic tree of *Wickerhamomyces subpelliculosus* ZE75 with other species in the GenBank database was shown in S_2_ (supplementary file).

### Kinetics and optimization of ethanol production by W.subpelliculosus ZE75

#### Effect of substrate on ethanol production and cell growth

The specific ethanol production rate (gg^−1^ h^−1^) reached the maximum value 0.138 using glucose as sole carbon source compared with the use of fructose 0.129, sucrose 0.132, lactose 0.0443 and maltose 0.0459. Ethanol yield using glucose was the greatest 0.598 (gg^−1^); however, biomass yields for both glucose and fructose substrates did not present a significant difference. In addition, a volumetric ethanol productivity of 1.247 gL^−1^ h^−1^ was achieved using glucose as the initial substrate. This productivity decreased significantly when another carbon source was used (Table 3).

**Table 3:** Effect of different carbon sources on ethanol and biomass yields by *W. subpelliculosus* ZE75

Parameters	Sucrose	Glucose	Fructose	Lactose	Maltose
Ethanol, *E*_*p*_ (gL^−1^)	78.50^a^ ± 0.16	89.77^b^ ± 0.17	82.83^c^ ± 0.11	13.78^d^ ± 0.21	11.89^e^ ± 0.35
Biomass, *X*_*m*_ (gL^−1^)	8.274^a^ ± 0.05	9.044^b^ ± 0.31	8.93^b^ ± 0.12	3.24^c^ ± 0.07	2.698^d^ ± 0.01
Volumetric ethanol productivity, *Q*_*p*_ (gL^−1^h^−1^)	1.0902	1.247	1.150	0.1435	0.1239
Specific ethanol production rates, *v*_*g*_ (gg^−1^h^−1^)	0.132	0.138	0.129	0.0443	0.0459
Ethanol coefficient yield, *Y*_*P/S*_ (gg^−1^)	0.5233	0.598	0.5522	0.0656	0.6606
Biomass coefficient yield, *Y*_*X/S*_ (gg^−1^)	0.0552	0.066	0.0596	0.0732	0.0761
Average sugar uptake, *Q*_*s*_ (gL^−1^h^−1^)	1.708	2.06	1.689	1.117	1.104

Values are means of three replicates; values followed by same letters on the same row are not significantly different (P < 0.005) in Tukey’s test

#### Effect of initial glucose concentration on ethanol production

In shake-flask experiments, kinetic studies were performed using different initial glucose concentrations (50–250 gL^−1^) at specific fermentation times. For each time interval, the variations in ethanol production concentration and dry biomass were determined. Different initial glucose concentrations were chosen as independent variable for the specific growth and ethanol production rates of the culture. The growth data was represented by Monod model as following:

7 $$\mu = \frac{{\mu_{\max } S_{Go} }}{{K_{s} + S_{Go} }}$$

where *µ*_max_ is the maximum specific growth rate, S_Go_ is initial glucose concentration, *K*_s_ is glucose utilization constant of the Monod model.

**Fig. 2:**
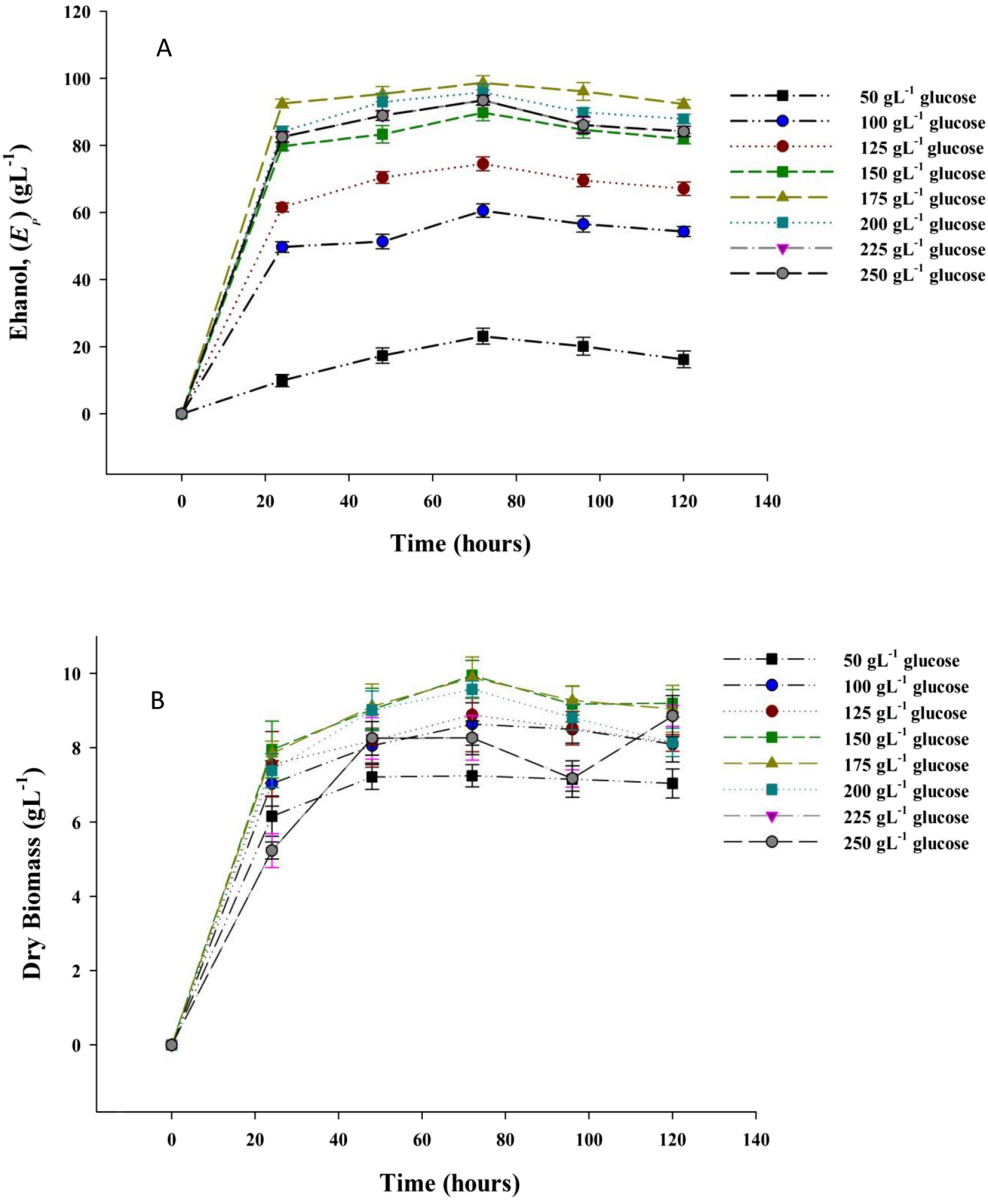
Variation in **a** Biomass and **b** ethanol production of *W. subpelliculosus* ZE75 on glucose during different fermentation periods.

The results obtained are shown in Fig. 2, showed that ethanol yield increased along with the increase in glucose concentration and reached the maximum ethanol production 98.65 gL^−1^ at sugar concentration of 175 gL^−1^ with ethanol productivity of 1.37 gL^−1^ h^−1^. The ethanol production reached a saturation limit when fermenting at a glucose concentration of 17% then. The maximum specific ethanol production and growth rates for the marine isolate were correlated using non-linear regression method as following:

$$v = 0.482\frac{{ S_{Go} }}{{469.69+ S_{Go} }},\;R^{2} = 0.951$$

$$\mu = 0.141\frac{{ S_{Go} }}{{24.40 + S_{Go} }},\;R^{2} = 0.912$$

**Fig. 3:**
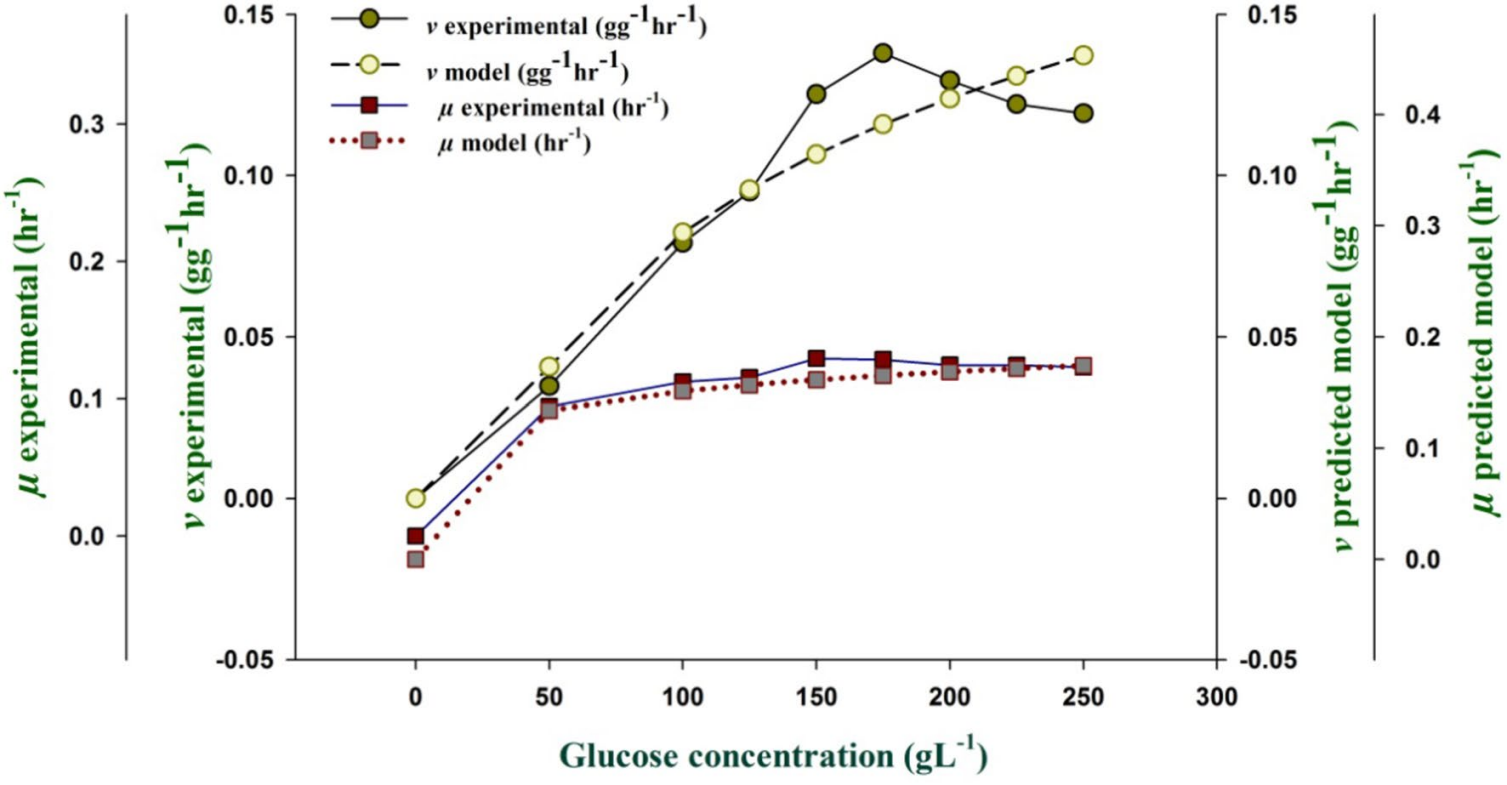
The experimental and model predicted specific growth and ethanol production rates by *W. subpelliculosus* ZE75 at different glucose concentrations

It was estimated that the maximum specific ethanol production rate (*v*_*max*_) and maximum growth specific rates (*μ*_max_) were calculated as 0.482 gg^−1^ h^−1^ and 0.141 h^−1^(Fig. 3) with high accuracy giving *R*^2^ values of 0.95 and 0.91, respectively. The maximum specific ethanol production rate was obtained at an initial glucose concentration of 175 gL^−1^ whereas the maximum specific growth rate was achieved at an initial concentration of glucose (150 gL^−1^).

### Effect of salinity on ethanol production

**Fig. 4:**
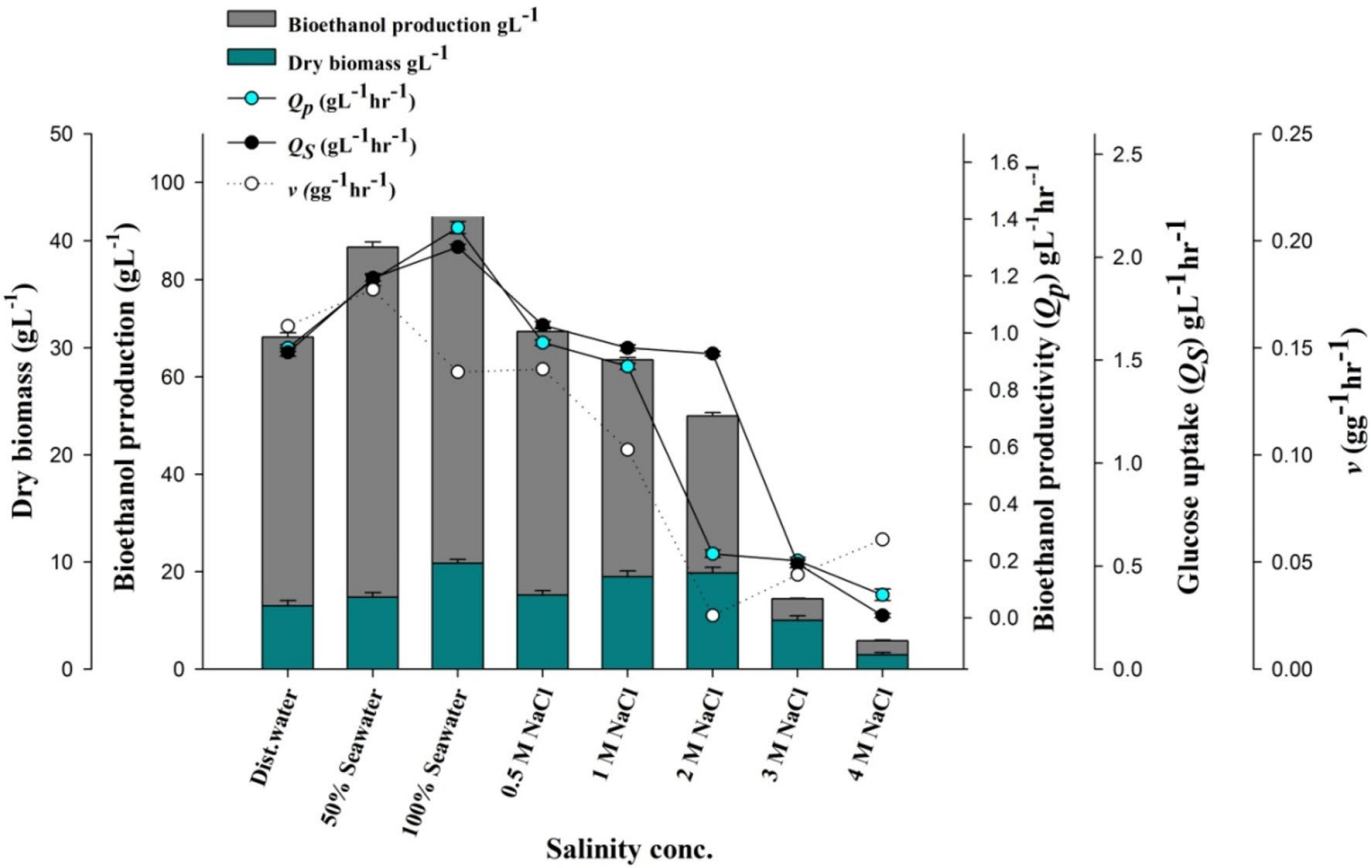
Effect of seawater, distilled water, 50% seawater and different salinity concentrations on ethanol and biomass production parameters

The effect of different salt concentrations, seawater, and distilled water on ethanol production was studied. The data in Fig. 4 showed that the highest ethanol yield 0.5637 gg^−1^ was achieved in a 100% natural seawater-based medium with a growth yield of 0.0561 gg^−1^. On the other hand, the isolate achieved a significant ethanol yield of 0.4954 in distilled water-based media with a growth yield of 0.0336 gg^−1^. Moreover, the marine isolate could grow and produce ethanol at sodium chloride concentrations up to 4 M, however, a marked decrease was observed in both ethanol and growth productivity proportional to the increase in salt concentrations.

### Effect of Effect of hydrogen ion concentration

**Fig. 5:**
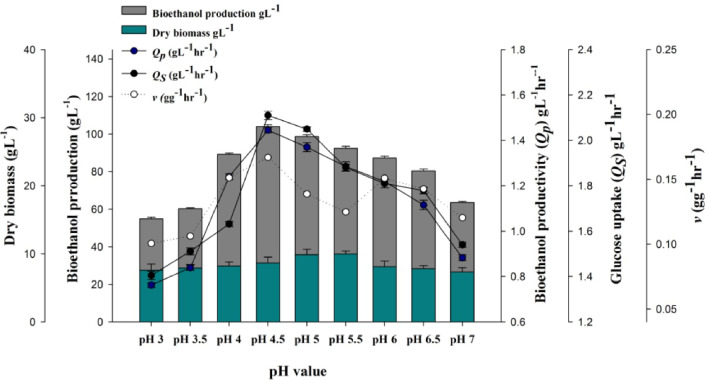
Effect of different pH values on ethanol and biomass production parameters

The resulted data in Fig. 5 revealed that the maximum ethanol production 104.04 gL^−1^ was achieved at pH 4.5 with specific ethanol rate of 0.1669 gg^−1^h^−1^. Moreover, the data showed a significant inhibition of ethanol production at pH 3 with ethanol production of 54.91 gL^−1^ and specific ethanol rate of 0.1006 gg^−1^ h^−1^.

### Comparison of ethanol production in this study with previously reported studies

Data in Table 4 showed a comparison of the ethanol production in the current study with previously reported studies by some marine yeast isolates using the batch culture technique. Based on the comparison table, it was shown that our isolate has a high ethanol productivity of 1.445 gL^−1^ h^−1^ compared to the previously reported marine yeasts.

**Table 4:** Comparison of ethanol production in the current study with previously reported studies by some marine yeast isolates using batch culture technique

Organism	Ethanol production *E*_*p*_ (gL^−1^)	Ethanol coefficient yield *Y*_*P/S*_ (gg^−1^)	Ethanol productivity *Q*_*p*_ (gL^−1^h^−1^)	Reference
*Candida albicans*	47.30	–	0.4927	Senthilraja et al. (2011)
*Pichia salicaria*	38.0	–	0.3958	Senthilraja et al. (2011)
*S. cerevisiae* HK21	70.0	0.466	0.5833	Urano et al. (2017)
*S. cerevisiae* AZ118	67.65	0.2706	0.99	Zaky et al. (2018)
*S. cerevisiae* AZ65	93.50	0.425	1.95	Zaky et al. (2018)
*W. anomalus* M15-500A	92.70	0.4635	0.4828	Turner et al. (2022)
*W. subpelliculosus* ZE75	104.04	0.5945	1.445	The current study

## Discussion

Marine environments have been considered as one of the most diverse and microbial-rich environments (Danovaro 2017). In the current study, screening of the marine yeast isolates (ZE2, ZE15, ZE68, ZE5, and ZE102) for ethanol production indicated that all the tested isolates could produce ethanol at variable concentrations using seawater-based medium. Seawater could be an interesting alternative to fresh water for ethanol production; it is sustainable, abundant, and contains a variety of minerals (Zaky et al. 2016). There is no longer a need to recycle water while using seawater. Moreover, distilled water can be considered an additional product during ethanol distillation (Zaky et al. 2020). However, seawater contains a high salt concentration (about 35 gL^−1^) along with various inhibitors (Greetham et al. 2019a, b). Marine yeasts can be an ideal choice for bioethanol production using seawater-based media. They are distinctive in fermenting sugars under high salt concentrations and have extraordinary tolerance to extreme environments (Connell et al. 2008; Hawary et al. 2019). These unique characteristics provide a special capability for the bioethanol production industry, especially in coastal areas where freshwater is priceless (Zaky et al. 2018).

The marine isolate ZE75 was selected for further studies. The Morphological characteristics of the selected isolate were matched to *Wickerhamomyces subpelliculosus* characterized by Kurtzman et al (2011) as budding, ascomycetous yeast, forming long, branching pseudohyphae and its asci have one to four hat-shaped ascospores. The physiological and biochemical characteristics (S1, supplementary file) revealed that the isolate ZE75 is ascomycetous yeast relying on its negative reaction to the diazonium blue B test (DBB). This test is carried out to determine whether asexual yeast is a basidiomycetous genus (+ve reaction) or belongs to ascomycetous yeasts (−ve reaction) (Kurtzman et al. 2011). Results also showed that the isolate has overall good sugar utilization capabilities in a seawater-based medium and grows under extreme stress conditions such as high osmotic pressure and vitamin-free medium. Zaky et al. (2014) reported that many marine yeasts have the capacity to utilize several substrates and tolerate stressful conditions. The high metabolism capacities of the isolated yeasts might be since the constant exposure to extreme environmental conditions such as salinity, low soluble carbon, and nitrogen levels, and other inhibitors that exist in seawater force the evaluation of new adaptive strategies and the synthesis of new metabolites (Rasmey et al. 2020).

*W. subpelliculosus* ZE75 could ferment different types of substrates and produce ethanol at variable concentrations. Leandro et al. (2011) revealed that marine yeasts could ferment a variety of substrates, sugars, and carbon sources.

The specific ethanol production rate reached the maximum value using glucose as the initial substrate. This may be because glucose is considered the most used energy source in cells (Xiao et al. 2014). Yeast strains could assimilate glucose extensively, whereas some showed low assimilation of sucrose and this variation may be due to their preference towards certain carbon sources which is directly correlated to the structure of hexokinase encoded sequences (Madzak 2021). Orlic et al. (2010) revealed that at high monosaccharide medium more DHAP and NADH were generated. Similar results were obtained by Jasman et al. (2015), who studied the ability of some yeast strains to consume sugars (sucrose, glucose, and fructose) and convert them into ethanol during fermentation, and reported that yeasts were capable of converting glucose into ethanol more efficient than fructose and sucrose and the ability of each strain to consume sugar and producing ethanol was different according to the genetic and physiologic stability of each yeast strain.

The obtained results in the current study were consistent with Dı´az-Nava et al. (2017) using the non-*Saccharomyces* yeast *Pichia kudriavzevii* ITV-S42, observed that biomass production of both glucose and fructose had no appreciable difference; however, the maximum ethanol production 1.24 (gg^−1^ h^−1^) was obtained when glucose was used as substrate with significantly lower compared to fructose 87.0. Although the metabolic flux in glucose fermentation is like that of fructose, the primary factors in the fermentation difference between glucose and fructose is the sugar transport through the membrane and the various steps of phosphorylation. Fructose is phosphorylated by HXK1 and HXK2, while glucose transport is phosphorylated by three enzymes, GLK1, the HXK1, and HXK2 (Colville et al. 1993). Another significant factor in transport is the physicochemical properties of substrates; fructose is transported in furanose and glucose in pyranose (Dı´az-Nava et al. 2017).

The integration of experimental studies with kinetic modeling can provide new aspects of microbial physiology and enable us to evaluate and predict the effects of changing the components of a fermentation process (Almquist et al. 2014). Monod kinetics models can be used to interpret product formation and biomass growth with respect to substrate utilization (Imamoglu and Sukan 2013).

It was observed that a decline effect was observed at initial glucose concentrations above the optimum substrate concentrations necessary for yeast cell growth and ethanol production. This might be because a sugar suppression of enzymes in the glycolytic flux fermentative metabolism switched and caused a slower conversion rate (Rasmey et al. 2018). Very high sugar concentrations might inhibit fermentation due to increasing osmotic stress (Timmermans et al 2022). Moreover, very high concentrations of sugar lead to an increase in the viscosity of the fermentation medium which has an inhibitory effect on yeast metabolism, sugar utilization, and the ability to produce ethanol (Zohri and Mostafa 2000; Reddy and Reddy (2006); . Our results were consistent with Chang et al. 2018, who reported that a considerable inhibitory effect on yeast growth, ethanol yield, and ethanol concentration was revealed when initial glucose concentrations were higher than 18% (w/v). Mauricio and Salmon (1992) demonstrated that a major limiting factor in fermentative metabolism is the inhibition of sugar transporters that have a specific affinity for glucose. Those transporters show a high affinity to the substrate which results in catabolic repression; however, they are not detected in fermentation at high sugar concentrations.

Chi et al. (2010) defined marine yeasts as yeasts that are isolated from marine habitats and grow best in seawater rather than a freshwater-based medium. The data showed that the highest ethanol yield was achieved in a 100% natural seawater-based medium. Seawater contains a wide range of minerals that could obviate the addition of other elements necessary to the commercial fermentation medium (Lin and Tanka 2006). Thus, the use of marine isolates in industrial fermentation processes can enhance the overall economy of the process. A marked decrease in both ethanol and growth productivity was observed proportional to the increase in salt concentrations. This may be due to the disproportionate skew of energy and carbon in cellular osmolyte production to maintain metabolic flux generated by high NaCl concentrations (Fernanda et al. 1999).

In addition to substrate concentration, pH is also a crucial factor that affects ethanol production (Liu et al. 2015). The hydrogen ion concentration influences the growth and fermentation rate of yeast and affects the constitution of fermentation products (Arroyo-López et al. 2010). The effect of different initial pH values on ethanol fermentation was investigated. Yaçlin and Ӧzbas (2008) reported that most yeast isolates can grow very well between pH 4.5- 6.5, and almost all species can grow in a more acidic or alkaline medium where low or high pH values ​​may cause chemical stress on the yeast cell, which is consistent with the results presented in the current study. The results obtained in this study were in accordance with the studies of Lin et al. (2012) and Reddy and Reddy (2011). A significant inhibition of ethanol production at pH 3 was observed. Low pH has a significant impact on lipid regulation, plasma membrane integrity, and perturbation of the function of proteins embedded in the cellular membrane, and thus cell growth and alcoholic fermentation are inhibited (Liu et al. 2015).

Ethanol production via marine yeasts is an attractive approach that can produce significant amounts using a seawater-based medium (Zaky et al. 2018). However, the selection of yeast strains and optimization of fermentation conditions are critical parameters for ethanol overproduction (Senthilraja et al. 2011; Zaky et al. 2020).

A comparison of ethanol production in this study with previously reported studies showed that our isolate has promising features for ethanol production.

## Conclusion

Ethanol production from marine yeasts can have a huge impact on overcoming both the fuel and freshwater crises. It was observed that the substrate type, initial concentration, and fermentation period are significant factors that affect ethanol production and yeast growth. Glucose is the best substrate for ethanol production by *Wickerhamomyces subpelliculosus* ZE75. Other fermentation parameters such as salinity and pH had a critical influence on ethanol production. The highest ethanol yield was achieved in a 100% natural seawater-based medium. The maximum ethanol productivity was achieved at pH 4.5. The present study revealed the ability of marine yeasts to produce ethanol in high yield from carbon sources dissolved in seawater.

### Supplementary Information

Below is the link to the electronic supplementary material.

Supplementary file1 (DOCX 184 kb)

## Data Availability

ITS-rDNA gene sequence (583 bp) of the isolate was deposited to the GenBank database, The novel isolate was deposited under the accession number OP715873 in the GeneBank. All data used has been included in the manuscript [and its supplementary information files].
