# Supplementary material for: Kinetic modeling and optimization of ethanol fermentation by the marine yeast Wickerhamomyces subpelliculosus ZE75
Source: World J Microbiol Biotechnol. 2024 Apr 6;40(5):155. doi: 10.1007/s11274-024-03942-y (PMC10998816; doi:10.1007/s11274-024-03942-y)
Supplement: Supplementary file 1 — Supplementary file1 (DOCX 184 kb) [file 11274_2024_3942_MOESM1_ESM.docx]

**S_1_.** Physiological and biochemical characteristics of *W. subpelliculosus* ZE75.

| Characteristic | Result |
| --- | --- |
| DBB | - |
| Citrate | - |
| MR | - |
| VP | - |
| Indole | - |
| Urease | - |
| Starch hydroysis | - |
| Celluolse hydrolysis | + |
| Vitamin free | + |
| 50% glucose | + |
| Fermentation: |  |
| Glucose | + |
| Sucrose | + |
| Maltose | + |
| Lactose | + |
| Galactose | - |
| Assimilation: |  |
| Glucose | + |
| Sucrose | + |
| Maltose | + |
| Lactose | + |
| Galactose | - |
| D- Xylose | - |
| L- Arabinose | + |
| Erythritol | + |
| Cellobiose | + |
| Nitrate | + |
| Glycerol | + |
| Temperature profile: |  |
| 4^o^C | - |
| 8^o^C | + |
| 12^o^C | + |
| 25^o^C | + |
| 30^o^C  37^o^C | +  + |
| 42^o^C | + |
| Halo-tolerance profile: |  |
| 0.5 M NaCl | + |
| 1 M NaCl | + |
| 2 M NaCl | + |
| 3 M NaCl | + |
| 4 M NaCl | + |

##
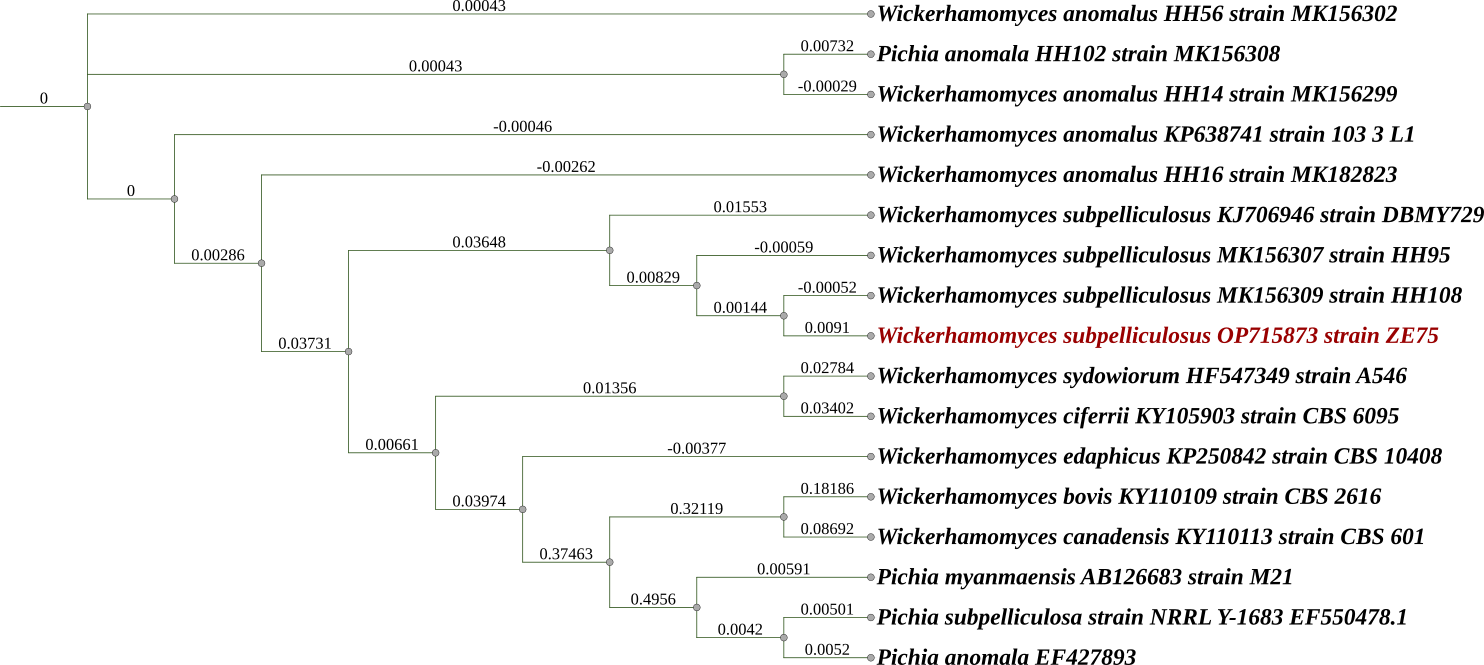


S_2_. Evolutionary relationships of isolated yeast strain *W. subpelliculosus* ZE75 with other species in GenBank database.
